# Fascia Lata Alterations in Hip Osteoarthritis: An Observational Cross-Sectional Study

**DOI:** 10.3390/life11111136

**Published:** 2021-10-25

**Authors:** Ilaria Fantoni, Carlo Biz, Chenglei Fan, Carmelo Pirri, Caterina Fede, Lucia Petrelli, Pietro Ruggieri, Raffaele De Caro, Carla Stecco

**Affiliations:** 1 Orthopedics and Orthopedic Oncology, Department of Surgery, Oncology and Gastroenterology (DiSCOG), University of Padua, 35128 Padua, Italy; ilaria.fantoni@studenti.unipd.it (I.F.); carlo.biz@unipd.it (C.B.); pietro.ruggieri@unipd.it (P.R.); 2 Department of Neurosciences, Institute of Human Anatomy, University of Padua, 35121 Padua, Italy; chenglei.fan@studenti.unipd.it (C.F.); caterina.fede@unipd.it (C.F.); lucia.petrelli@unipd.it (L.P.); rdecaro@unipd.it (R.D.C.); carla.stecco@unipd.it (C.S.)

**Keywords:** fascia, hip osteoarthritis, hyaluronan, collagen, stiffness, myofascial pain

## Abstract

The present study compares the structure and composition of fascia lata in healthy subjects and in patients with hip osteoarthritis (OA), to evaluate any differences in the amount of Collagen type I, Collagen type III, and Hyaluronan. Fascia lata samples from voluntary healthy subjects and patients with OA were harvested during surgery. Collagen type I (COL I), III (COL III) antibody, and biotinylated hyaluronan binding protein (HABP) immunohistochemistry stainings were used to evaluate fascial morphology and COL I, COL III, and Hyaluronan (HA) content in both groups. Ten samples from healthy subjects and 11 samples from OA patients were collected. COL I was significantly more abundant in the OA group (*p =* 0.0015), with a median percentage positivity of 75.2 (IQR 13.11)%, while representing only 67 (IQR: 8.71)% in control cases. COL III, with median values of 9.5 (IQR 3.63)% (OA group) and 17.10 (IQR 11)% (control cases), respectively, showed significant reduction in OA patients (*p =* 0.002). HA showed a median value of 10.01 (IQR 8.11)% in OA patients, denoting significant decrease (*p <* 0.0001) with respect to the control group median 39.31 (IQR 5.62)%. The observed differences suggest a relationship between fascial pathology and hip OA. The observed increase in COL I in OA patients, along with the reduction of COL III and HA, could lead to fascial stiffening, which could alter fascial mechanics and be linked to the development and symptoms of OA.

## 1. Introduction

Osteoarthritis (OA) is a degenerative pathology of the joint [1], characterized by progressive damage to articular cartilage, which becomes brittle and tends to fragment [2], with subsequent joint space narrowing and reactive bone alterations such as osteophytes, cysts, and subchondral bone sclerosis [3]. OA currently represents an important cause of disability [4], with an estimated prevalence ranging from 1 to 10% in the general population [5,6,7,8]. It is more common in female subjects [9] and in the elderly [10,11]. 

It can be secondary to pre-existing conditions leading to structural alterations of the joint, such as congenital diseases [4], traumas, metabolic or endocrine diseases, infections, and avascular necrosis [2]. Most cases, however, can be referred to as primary forms, whose aetiology is unknown, despite the high prevalence of OA [4]. It is considered multifactorial, with contribution of both endogenous factors, as age, sex, heredity [2,12], and exogenous factors, including physically demanding activities [13], obesity, and cigarette smoke [14]. 

Up to now, no treatment have been proven to stop or delay satisfactorily the progression of the disease [15]. Nonoperative treatment, mainly aimed at symptoms alleviation, comprises weight loss [16,17], low-impact aerobic exercise [14,18,19], physical therapy [20], pharmacologic treatments with Acetaminophen, Non-steroidal Anti-inflammatory drugs (NSAIDs) and opioid analgesics [21], intra-articular corticosteroid injections [22,23] and viscosupplementation with hyaluronic acid [24,25,26,27]. Joint arthroplasty is currently the treatment of choice after failure of nonoperative measures [18]. 

Despite several mechanisms having been proposed to explain its onset and progression, the aetiopathogenesis of osteoarthritis is still doubtful. Recent literature focuses on intra-articular environment, evaluating chondrocyte or synovial alterations, while little interest has been reserved to extra-articular structures. In particular, the role of fascia in the pathology is still unclear, as a characterization of fascial morphology and composition in hip OA is currently lacking, although fascial alterations have already been related to several musculoskeletal pathologies, as non-specific low back pain, sacro-iliac joint pain, and chronic shoulder pain [28,29,30,31]. Fascia is a lamina of connective tissue dissectible through a cleavage plane [32], defining a three-dimensional tensional network throughout the body [33]. It shows different features and specializations according to the anatomical region, aimed at optimizing its interaction with adjacent structures [34]. It surrounds and separate muscles, allowing the transmission of muscular force between body segments [35], it strengthens capsular and ligamentous structures, it forms the connective neuro-vascular sheath around vessels and nerves, the periosteum over bones, the paratenon over tendons [29]. Fascia lata is the deep/muscular fascia of the thigh, that have an important role in transmission of the force and load in the lower limb. Its relationship with the muscles of the hip is crucial in the hip biomechanics. Indeed, for example, the superficial fibres of the gluteus maximus muscle insert into the iliotibial tract and the lateral intermuscular septum that connects with the fascia lata [36]. 

Fascial composition and organization, responsible for fascial properties [31], can be modified by several stimuli. Even if it is not influenced by age nor by sex [37], variations in the composition of extracellular matrix have been shown after stimulation of the endocannabinoid system [38] and after changes of estrogen levels [39].

Moreover, Fede et al. demonstrated that fasciae are rich in hyaluronan (HA), which guarantees the gliding ability of the fasciae in respect to the adjacent tissues. The amount of HA varies according to the anatomical site and fascial function, showing higher concentration in aponeurotic fasciae, particularly in the retinacula, which do not adhere to underlying muscle, thus, being able to glide over them [37]. Changes in the amount of HA could have an important role in the development of fascial dysfunctions [40]. Indeed, variations in its concentration could alter the density, and, thus, the viscoelasticity, of the extracellular matrix [41]; these changes could affect fascial gliding, impairing muscle and joint biomechanics, with consequent pain and loss of function [35]. Fascial alterations have recently been linked to the development of myofascial pain. Indeed, they could have a role in pain perception due to their rich innervation by a network of small nerve fibres [42]. 

This study hypothesizes an association between fascia lata alterations and hip OA; in particular, changes in the production of collagen and HA could result in altered fascial structure and behaviour in OA patients.

Hence, this research is aimed at comparing the content of type I collagen, type III collagen, and HA between healthy subjects and patients with hip OA, in order to understand if fascia lata composition can be altered in OA cases.

## 2. Materials and Methods

The present research was approved by the Istitutional Ethical Committee (n. 3722/AO/16) and performed in accordance with the ethical standards of the 1964 Declaration of Helsinki as revised in 2000 and those of Good Clinical Practice. All subjects participating in the study received a thorough explanation of the risks and benefits of inclusion and gave their oral and written informed consent to publish the data.

Patients undergoing hip arthroplasty, either following hip OA or femoral neck fracture, or intertrochanteric hip fixation at the Orthopaedic Clinic of the University of Padua were included in the study. Exclusion criteria were malignant neoplasms, previous hip surgery, acute inflammatory diseases, or infectious diseases. 

For each patient, the presence and severity of hip osteoarthritis were evaluated on pre-operative X-rays according to the Kellgren–Lawrence classification [43]. Patients were then divided in two groups depending on radiological findings: group A included patients with grade 0 to 1, showing absent or minimal signs of OA, while group B comprised subjects with mild to severe OA, graded 2 to 4 [44]. All patients reported to be able to walk and self-sufficient before trauma; however, a preoperative functional assessment of the affected hip could not be performed, as the majority of the evaluated cases were trauma patients.

For each patient, during surgery, a 1 cm^2^ sample was harvested from the anterolateral portion of fascia lata, proximal to the greater trochanter. All specimens were immersed in phosphate buffered saline and transported to the laboratory within less than one hour since harvest.

After formalin fixation, samples were dehydrated in graded ethanol, embedded in paraffin, and cut into 5 mm-thick sections. On dewaxed and hydrated sections, Hematoxylin and Eosin (H&E) and Picrosirius stainings were performed for histological analysis.

### 2.1. Immunohistochemistry

After deparaffination and hydration, sections were treated with a solution of H_2_O_2_ in order to inhibit endogenous peroxidase activity. For collagen I (COL I) and collagen III (COL III), after three washings in phosphate buffered saline (PBS), samples were incubated with bovine serum albumin (BSA) 0.2%. They were then treated overnight with the primary antibody (Goat Anti-Collagen I, 1:400, Southernbioteck; Rabbit polyclonal to Collagen III, 1:400, ab7778AbCam, respectively) in BSA. After repeated washings in PBS, the sections were incubated with the secondary antibody (anti-goat peroxidase-conjugated antibodies 1:300 for Collagen I; anti-rabbit peroxidase-conjugated antibodies for Collagen III 1:200, Jackson Immunoresearch, Cambridgeshire, UK). After three washings in PBS, the reaction was developed with 3,3′-diaminobenzidine (Liquid DAB + Substrate Chromogen System; Dako). Samples were finally counterstained with Hematoxylin, dehydrated, and mounted with a coverslip using Eukitt (Agar Scientific). For each staining, a negative control was obtained by omitting the primary antibody.

To assess the amount of hyaluronan, an immunohistochemical staining for Hyaluronan Binding Protein (HABP) was performed. After deparaffination and hydration, samples were treated with avidin and biotin solutions (Biotin-Blocking System, Dako, Carpinteria, CA, USA) to block any endogenous avidin biotin activity. Sections were then incubated with a solution of 0.5% H_2_O_2_ to block any endogenous peroxidase activity. They were then washed with 0.2% Triton-X, in PBS and incubated in 0.2% bovine serum albumin, BSA. Sections were then treated overnight with biotinylated HABP, 2 mg/mL (Millipore), in the same pre-incubation buffer, 1:900 dilution. After repeated washings in PBS, samples were incubated with the secondary antibody (HRP conjugated Streptavidin 1:250, Jackson ImmunoResearch, Cambridgeshire, UK). After three washings in PBS, the reaction was developed with 3,3′-diaminobenzidine (Liquid DAB Substrate Chromogen System, Dako). Procedure was completed with Hematoxylin staining, dehydration in graded ethanol series and coverslip mounting with Eukitt. Negative controls were checked with similarly treated sections, omitting the primary antibody.

### 2.2. Image Analysis

Specimen pictures, taken with a digital camera (Leica Microsystems, Wetzlar, Germany) were analyzed using ImageJ software [45]. At a magnification of 5×, the percentage antibody positivity for COL I, COL III, and HABP were obtained. At least five pictures from five sections for every specimen were counted. Data obtained from the analysed images were averaged to obtain the representative values. Results were expressed as percentage antibody positivity per unit area (where the unit area was equivalent to the field covered at 5× magnification). The image analysis was made by a single reader in different times. We calculated the intra-reader reliability by Intra-class-correlation coefficient, ICC_2,k_: 0.9 (0.85–0.95).

### 2.3. Statistical Analysis

Data were analysed using IBM SPSS version 25.0 software (SPSS Inc., Chicago, IL, USA). The resulting effect size was calculated by G Power 3.1 according to Cohen’s d [46] and interpretated as small (d = 0.20), medium (d = 0.50), and large (d = 0.80). For collagen 1, the effect size was d = 1.63 in a first our unpublished study, α err prob = 0.05, power: 1-β err prob = 0.95; sample size was for group = 9. The Kolmogorov–Smirnov and Shapiro–Wilk tests were used to evaluate data distribution; Levene test evaluated the homogeneity of variance test. Percentage content per unit area was reported as median ± interquartile range (IQR). Each value was analysed with Mann–Whitney test for independent values, in order to compare Group A and B. Significance was settled for *p* ≤ 0.05.

## 3. Results

### 3.1. Sample Characteristics

Twenty-one patients were recruited for the research, seven males and 14 females, with a mean age of 86 ± 11.68 years. Group A was composed by 10 patients, four males and six females, with a mean age of 76.9 ± 12 years. Group B included 11 patients, three males and eight females, showing a mean age of 85.82 ± 10.06 years. Sample characteristics are represented in Table 1.

### 3.2. Immunohistochemical Analysis

**Figure 1:**
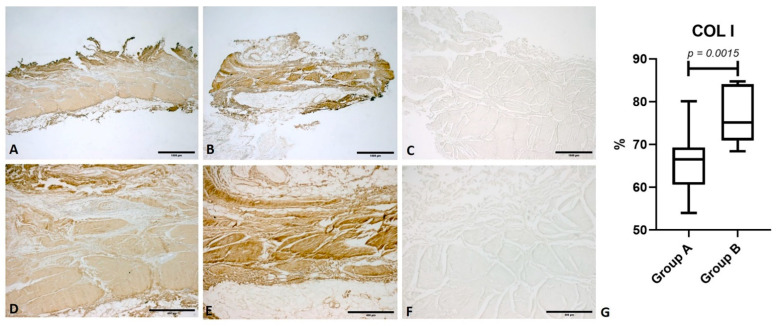
Immunostaining for collagen I (COL I). Comparison between fascia lata samples from a healthy subject (Group A) and a patient with osteoarthritis (Group B). (**A**) Group A: fascia lata (1× magnification); (**B**) Group B: fascia lata (1× magnification); (**C**) Negative control: omission of the primary antibody (1× magnification); (**D**) Group A: fascia lata (5× magnification); (**E**) Group B: fascia lata (5× magnification); (**F**) Negative control (5× magnification); (**G**): comparison of the percentage antibody positivity for COL I.

In both groups, image analysis showed diffuse antibody positivity for collagen I (COL I) in the parallel fibrous fascicles of fascia lata. The amount of COL I was increased in group B, with a median percentage antibody positivity of 75.2 (IQR 13.11), while group A showed a median percentage of 67 (IQR: 8.71). There was statistical significance between the two groups (*p* = 0.0015) (Figure 1).

**Figure 2:**
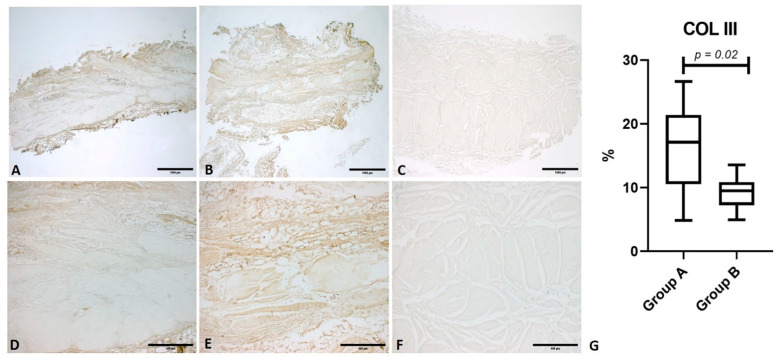
Immunostaining for collagen III (COL III). Comparison between fascia lata samples from a healthy subject (Group A) and a patient with osteoarthritis (Group B). (**A**) Group A: fascia lata (1× magnification); (**B**) Group B: fascia lata (1× magnification); (**C**) Negative control: omission of the primary antibody (1x magnification); (**D**) Group A: fascia lata (5× magnification); (**E**) Group B: fascia lata (5× magnification); (**F**) Negative control: omission of the primary antibody (5× magnification); (**G**): comparison of the percentage antibody positivity for COL III.

Collagen III (COL III) showed a median percentage positivity of 17.10 (IQR 11)% in group A; among arthritic patients it was significantly reduced (*p* = 0.02), with a median value of 9.5 (IQR 3.63)% (Figure 2).

**Figure 3:**
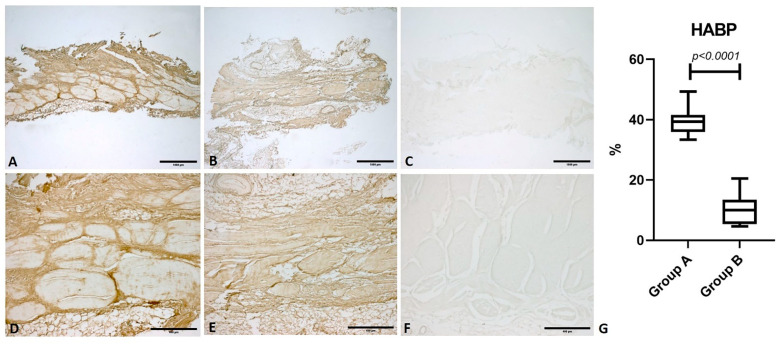
Immunostaining for Hyaluronan Binding Protein (HABP). Comparison between fascia lata samples from a healthy subject (Group A) and a patient with osteoarthritis (Group B). (**A**) Group A: fascia lata (1× magnification); (**B**) Group B: fascia lata (1× magnification); (**C**) Negative control: omission of the primary antibody (1× magnification); (**D**) Group A: fascia lata (5× magnification); (**E**) Group B: fascia lata (5× magnification); (**F**) Negative control: omission of the primary antibody (5× magnification); (**G**): comparison of the percentage antibody positivity for HABP.

Hyaluronan Binding Protein (HABP) showed a median percentage positivity of 39.31 (IQR 5.62)%. In group B, the median percentage was 10.01 (IQR 8.11)%. These data show a significant reduction of HA content in OA patients (*p <* 0.0001) (Figure 3). 

## 4. Discussion

Despite the diffusion of OA and the several proposed aetiologic mechanisms, most literature focuses on alterations of intra-articular environment, while the role of extraarticular structures in the development of the disease has been mostly overlooked. To our knowledge, no previous research has assessed any possible alterations of deep fascia in OA joints.

Data from immunohistochemical analysis showed that collagen type I is significantly increased in the fascia lata of patients with OA. This alteration could result in a more rigid fascia lata in patients with OA, as this molecule is the main component of the parallel bundles of dense connective tissue responsible of fascial stiffness and force transmission. Indeed, the increases in COL I content following mechanical or hormonal stimuli [38] have already been linked to the development of fascial stiffness and decreased range of motion [47].

Collagen III was mainly located at the periphery of collagen fascicles and in the loose tissue between adjacent fascicles. It showed significant reduction in OA subjects. Considering that this collagen type is present above all in the loose connective tissue and permits a better adaptability of the fascial tissue, its reduction can cause a stiffer fascia. Fede et al. [38] have already demonstrated that collagen types in fasciae change under hormonal stimulation, and that Collagen type III production is increased during pregnancy, to allow the necessary myofascial adaptations of the body to the new condition.

HA was concentrated in loose tissue between adjacent fibrous fascicles. It showed a significative reduction in cases with OA, as represented by the reduced positivity to HABP. Since this molecule, mainly located in the loose connective tissue between adjacent fascicles, is fundamental for the correct sliding of fascial planes [36,42], its alteration could further contribute to fascial stiffness and to the creation of a fascial densification.

The present study highlights for the first time a structural alteration of fascia lata in patients with hip OA. The observed increase of COL I, along with the reduction of COL III and HA, probably suggests a change in fascial mechanics, with stiffness and reduction of its physiological sliding properties, and the development of OA. A rigid fascia could indeed distort hip posture and gait, possibly resulting in joint overload, thus, contributing to disease pathogenesis and progression. Moreover, reduced fascial compliance could further limit joint range of motion, thus, worsening symptoms of OA as pain and movement reduction. Alternatively, the observed alterations could be a consequence of OA. In fact, altered joint mechanics subsequent to the disease could stimulate increased synthesis of COL I and decreased production of COL III and HA; the resulting altered composition could determine a fascial disfunction which could further worsen symptoms.

On the basis of the present study, it is not possible to discriminate whether the observed fascial changes can be a contributing factor in the pathogenesis of OA or if they should be regarded as a consequence of the disease. Further research, particularly focused on initial stages of the pathology, is needed to determine the causal relationship between fascial composition and hip OA.

Nevertheless, the correlation between fascial stiffness and hip OA can give several hints for the future treatment of OA. Firstly, it could give a rationale for nonoperative treatments aimed at treating fascial disfunction and restoring normal gliding properties of extraarticular tissues either in initial stages, where they could slow disease progression and reduce symptoms, and for patients not suitable for surgery, in which they could decrease pain and joint stiffness, thus, helping symptoms’ control. Despite that several studies [14,18,20] have concentrated on the role of muscle strengthening physiotherapy and exercise in the initial management of OA, to our knowledge no studies evaluate the effect of treatments acting on fasciae. Given the recognized role of fascia in the development of several musculoskeletal disfunctions and the observed structural alterations of arthritic fascia lata, a manual therapy aimed at assessing alterations in fascial gliding and at directing treatment specifically on dysfunctional points to restore normal fascial properties, for istance the Fascial Manipulation technique, could be a valuable tool to reduce pain and to improve movement and gait.

Besides, unrecognized fascial disfunctions, both preceding surgery or related to post-intervention fibrosis, could have a role in the persistence of pain and stiffness after hip arthroplasty, since they could maintain an altered limb mechanics, at least partly contributing to failure of surgical treatment. A proper detection of these conditions could, thus, allow proper treatment of these complications.

## 5. Study Limitations

The present research has several limitations.

First, age is different between the analysed group, with the OA group showing an average older age. As age can also determine a modification in the fascial ECM [48,49], it could have at least partly contributed to the observed differences in the two groups. However, from previous research, it has been demonstrated, for example, that the amount of HA shows no significant variation between adult and elderly people [36]. Furthermore, the two groups under comparison have a comparable advanced age, so our results are attributable to the advancement of the disease. Further data from a wider patient series are needed to improve our results.

Furthermore, we analysed only three components of the fascial ECM; study of other fascial ECM elements will be crucial to understand the complex multifactorial aspects of their alterations in hip osteoarthritis.

Finally, another limitation of this study is the lack of specific hip functional scores that took in account the fascial alteration and of a clear diagnostic to confirm and quantify the involvement of fasciae. Certainly, it is necessary to develop specific scales that consider fascial disorders to better characterize the role of this structure in hip osteoarthritis.

## 6. Conclusions

Hip OA is related to a structural alteration of fascia lata, which shows an increase in COL I content, along with a reduction in COL III and HA. These results suggest that hip OA is associated with a dysfunctional, stiffened fascia lata, with impaired sliding. An altered fascial behaviour could play a role in the pathogenesis of the disease and contribute to symptoms. Alternatively, fascial alterations could be stimulated by altered hip mechanics, and, thus, be a consequence of hip OA.

## Figures and Tables

**Table 1:** Descriptive data of the samples (Group A and B). y.: years old. BMI: body mass index.

Group A	Sex	Age (y.)	Height (cm)	Weight (kg)	BMI	Group B	Sex	Age (y.)	Height (cm)	Weight (kg)	BMI
1	F	73	180	80	24.69	1	F	90	184	72	21.27
2	F	62	172	69	23.32	2	F	83	185	80	23.37
3	F	91	170	70	24.22	3	F	92	168	65	23.03
4	M	89	172	62	20.96	4	M	90	175	79	25.80
5	M	56	180	83	25.62	5	F	94	182	65	19.62
6	M	68	185	78	22.79	6	M	59	175	93	30.37
7	M	89	175	80	26.12	7	F	86	176	89	28.73
8	F	84	185	82	23.96	8	F	91	177	84	26.81
9	F	78	168	70	24.80	9	F	88	177	84	26.81
10	F	79	183	76	22.69	10	M	93	177	77	24.58
						11	F	78	175	74	24.16
Mean ± SD		76.9 ± 12	177 ± 6.4	75 ± 7	24 ± 1.53	Mean ± SD		85.82 ± 10.1	177 ± 5	79 ± 9.24	25.33 ± 3.1

## Data Availability

The data presented in this study are available on request from the corresponding author.
